# The regulatory role of bile acid microbiota in the progression of liver cirrhosis

**DOI:** 10.3389/fphar.2023.1214685

**Published:** 2023-06-21

**Authors:** Feng Zhu, Shudan Zheng, Mei Zhao, Fan Shi, Lihong Zheng, Haiqiang Wang

**Affiliations:** ^1^ Heilongjiang University of Chinese Medicine, Harbin, China; ^2^ First Clinical School of Medicine, Heilongjiang University of Chinese Medicine, Harbin, China; ^3^ Department of Gastroenterology, Fourth Affiliated Hospital, Heilongjiang University of Chinese Medicine, Harbin, China; ^4^ Department of Gastroenterology, First Affiliated Hospital, Heilongjiang University of Chinese Medicine, Harbin, China

**Keywords:** bile acids, cirrhosis, gut microbiome, bile acids-gut microbiome axis, FXR, hepatic encephalopathy

## Abstract

Bile acids (BAs) are synthesized in liver tissue from cholesterol and are an important endocrine regulator and signaling molecule in the liver and intestine. It maintains BAs homeostasis, and the integrity of intestinal barrier function, and regulates enterohepatic circulation *in vivo* by modulating farnesoid X receptors (FXR) and membrane receptors. Cirrhosis and its associated complications can lead to changes in the composition of intestinal micro-ecosystem, resulting in dysbiosis of the intestinal microbiota. These changes may be related to the altered composition of BAs. The BAs transported to the intestinal cavity through the enterohepatic circulation are hydrolyzed and oxidized by intestinal microorganisms, resulting in changes in their physicochemical properties, which can also lead to dysbiosis of intestinal microbiota and overgrowth of pathogenic bacteria, induction of inflammation, and damage to the intestinal barrier, thus aggravating the progression of cirrhosis. In this paper, we review the discussion of BAs synthesis pathway and signal transduction, the bidirectional regulation of bile acids and intestinal microbiota, and further explore the role of reduced total bile acid concentration and dysregulated intestinal microbiota ratio in the development of cirrhosis, in order to provide a new theoretical basis for the clinical treatment of cirrhosis and its complications.

## 1 Introduction

The liver is a key hub for the crosstalk between host metabolism and gut microbiota, and the homeostasis of BAs and the micro-ecosystem plays an important role in maintaining human health (Shao et al., 2021). The host synthesizes primary BAs in the liver, which are subsequently converted to secondary BAs in the gut by the gut microbiota. BAs are a signaling molecule that mediates the liver-microecological axis and affects lipid homeostasis, inflammatory response, fibrosis, intestinal barrier function, and ultimately affects the severity of disease in patients with cirrhosis (Simbrunner et al., 2021). The complex relationship between bile acids and intestinal microbiota has been identified in several clinical trials, and elevated serum bile acid levels are strongly associated with reduced neurological function in cirrhosis and chronic liver disease (McMillin et al., 2016). Cirrhosis and chronic liver disease are associated with the ability of the intestinal microbiota to interfere with the composition of BAs and the size of the BAs pool, which gradually decreases with the severity of the loss of compensation. In addition, BAs are the main factor regulating intestinal microbiome, and if the concentration of BAs in the intestinal lumen decreases, it will lead to a decrease in beneficial flora and an increase in pathogenic bacteria in patients with cirrhosis, and dysbiosis of the intestinal microbiota may occur with the progression of cirrhosis and hepatic encephalopathy (HE). Therefore, regulation of BAs and intestinal flora has become a new strategy for the treatment of cirrhosis as well as its complications.

## 2 Bile acids and hepatic-intestinal circulation

### 2.1 Bile acids synthesis pathway

**FIGURE 1:**
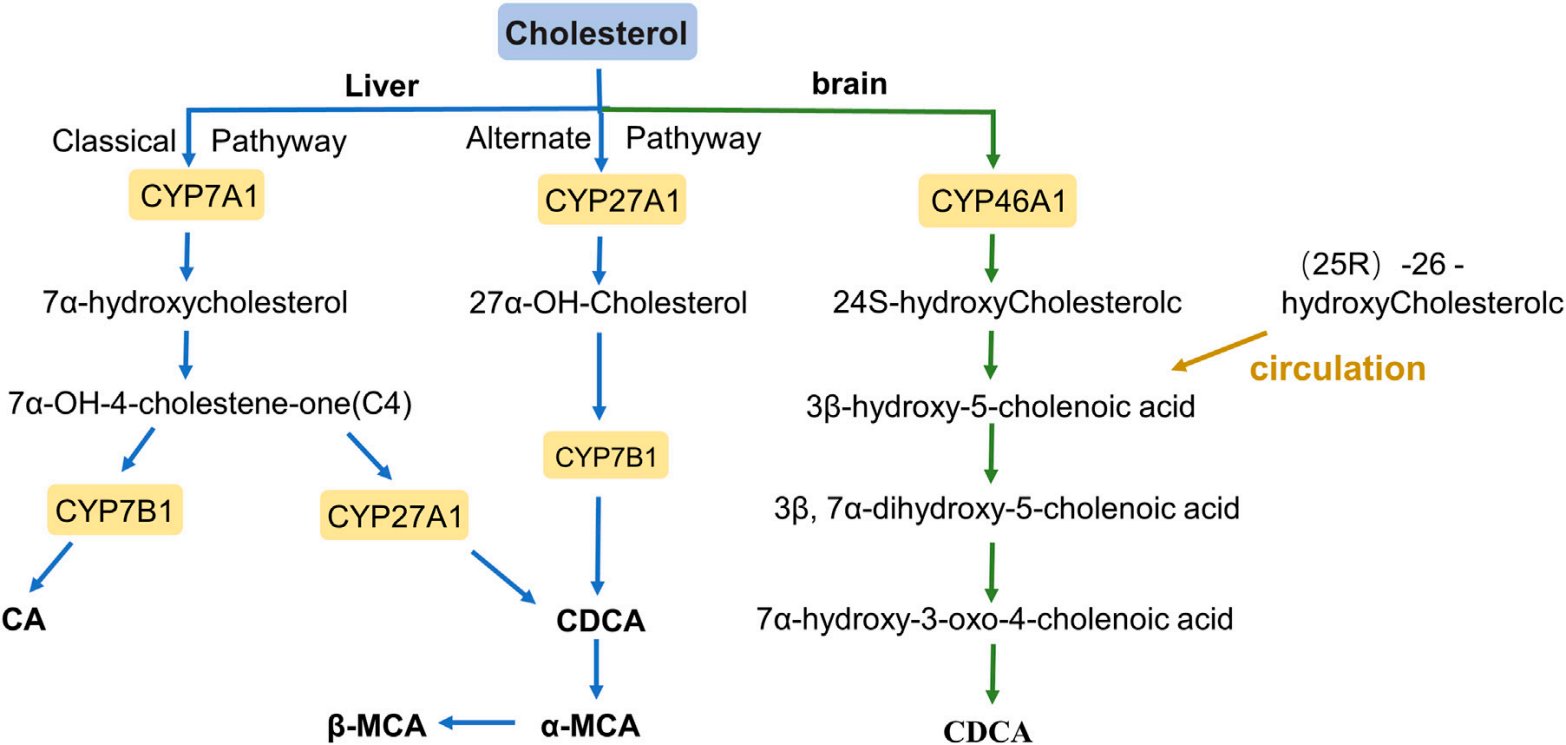
The bile acid synthesis pathway.

BAs are amphiphilic detergents, signaling molecules synthesized in the liver from cholesterol, and are synthesized mainly through two pathways: the classical pathway and the alternative pathway (Figure 1) (Chiang, 2009). The classical pathway mainly synthesizes Cholic acid (CA) and Chenodeoxycholic acid (CDCA), which account for more than 90% of the total bile acid pool in humans; therefore, the classical pathway is considered to be the main pathway for bile acid synthesis (Li and Chiang, 2014). BAs are first modified to form CA and CDCA by a microsomal cytochrome P450 7A1 (CYP7A1), cholesterol 7α hydroxylase, which is expressed only in the liver (Hofmann, 1984). CYP7A1 acts as the rate-limiting enzyme in the classical synthesis pathway of bile acids (Jelinek et al., 1990), a specific enzyme that converts cholesterol to 7α-hydroxycholesterol, which is then converted to 7α-hydroxy-4- cholesten-3-one (C4). C4 is a serum marker that reflects the rate of BAs synthesis in the liver, formed CA under the further action of cytochrome P450 8B1 (CYP8B1), or converted to CDCA by mitochondrial sterol 27-hydroxylase (CYP27A1) modification (Axelson et al., 1988; Russell and Setchell, 1992; Chiang, 2004; Chiang and Ferrell, 2020). In addition, the overall rate of bile acid synthesis is regulated by CYP7A1, and CYP8B1 regulates the CA/CDCA ratio in the bile acid pool (Li and Chiang, 2014).

**FIGURE 2:**
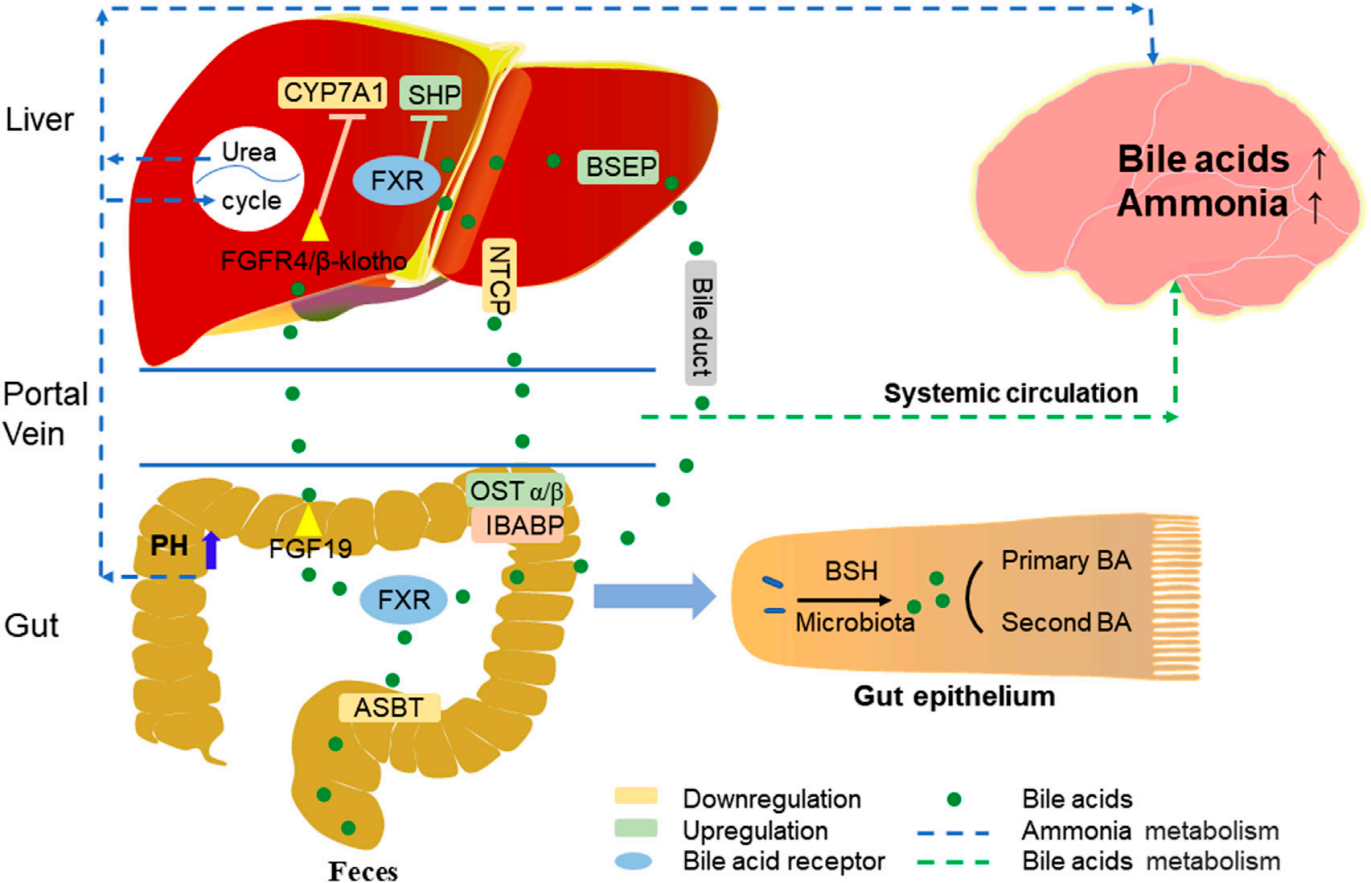
Bile acid signals enterohepatic-brain circulation.

In the alternative pathway, 27-hydroxycholesterol is oxidized from cholesterol via mitochondrial steroid 27-hydroxylase (CYP27A1) and then further hydroxylated by oxysterol 7α-hydroxylase (CYP7B1) to form CDCA, most of which is converted to α-muricholic acid (α-MCA) and β-MCA in rodents (Fakheri and Javitt, 2012). These BAs are conjugated to the glycine and taurine in hepatocytes tissues and are now referred to as primary BAs or conjugated BAs; glycine is the major BAs conjugate in humans, while BAs in rodents are mainly conjugated with taurine (Li and Apte, 2015; Gruner and Mattner, 2021). Primary BAs are synthesized in the hepatocytes, and stored in the gallbladder with bile through the tubular membrane. After eating, the duodenum secretes cholecystokinin to stimulate gallbladder contraction, which then releases bile acids into the intestinal tract (Li and Chiang, 2014). In the intestine, bacterial bile salt hydrolase (BSH) catalyzes the deconjugation, oxidation, and 7α-dehydroxylation of conjugated bile acids, modifying CA and CDCA in humans to secondary BAs, deoxycholic acid (DCA), and lithocholic acid (LCA), and ursodeoxycholic acid (UDCA), respectively (Figure 2) (Begley et al., 2005; Fu et al., 2012). In rodents, β-MCA forms ω-MCA via 6β-epimerase by the gut bacteria, LCA can also form hyodeoxycholic acid (HDCA) and murideoxycholic acid (MDCA) (Eyssen et al., 1983; Fu et al., 2012). In rodents, β-MCA forms ω-MCA via 6β-differential isomerization, and LCA can also be metabolized via dehydroxylation to form murideoxycholic acid (MDCA) and porcine deoxycholic acid (HDCA).

In the brains of rodents and humans, cholesterol 24 hydroxylase, a cytochrome P450 (CYP46A1), catalyzes the synthesis of 24S-hydroxycholesterol, androxylate which is converted to CDCA through the formation of intermediates by several enzymes. moreover, (25R)-26-hydoxycholesterol enters the brain in large quantities through the circulation and is likewise converted to 3β-hydroxy-5-cholenoic acid (Lund et al., 2003; Heverin et al., 2005; Kiriyama and Nochi, 2019).

### 2.2 Nuclear receptor of bile acids–FXR

FXR is a nuclear hormone receptor involved in glucose, energy and lipid metabolism and plays a central role in regulating BAS homeostasis, FXR is mainly expressed in the liver, ileum and kidneys (Stofan and Guo, 2020). BAs were recognized to be the endogenous ligands of FXR, CDCA is the most effective, followed by LCA, DCA, and CA. Activation of FXR can reduce the uptake of BAs in the intestine and the synthesis of BAs in the liver, and enhances biotransformation of BAs for export to bile to maintain low BAs levels in hepatocytes and prevent liver injury and cholestasis (Sauerbruch et al., 2021).

The effect of FXR on BAs has been demonstrated in FXR knockout (KO) mice, where both BAs synthesis and BAs pool size were increased in KO mice (Kok et al., 2003). FXR regulates many downstream signals, such as peroxisome proliferator-activated receptor (PPAR), Activation of FXR inhibits the progression of liver fibrosis, FXR ligands induce PPARγ gene expression in human hepatic stellate cells (HSCs), upregulates PPARγ mRNA in HSC and murine liver fibrosis models, and prevents downregulation of PPARγ caused by liver disease, PPARγ ligands inhibit HSC activation and enhanced antifibrotic activity (Pineda Torra et al., 2003; Fiorucci et al., 2005). It is worth mentioning that in the study of cirrhotic rats, folic acid can reverse the reduction of FXR signals in the ileum, improve the state of the mucus layer, and stabilize the intestinal vascular barrier. This conclusion supports the involvement of nuclear receptor FXR in regulating the intestinal vascular and mucosal barrier in cirrhosis (Sorribas et al., 2019).

### 2.3 Bile acid transporters with enterohepatic circulation

#### 2.3.1 BAs transporters in the intestinal and hepatic circulation

Most BAs absorption in the liver, as well as reabsorption in the intestine, relies on active transport across cell membranes, and the circulation of BAs in the gut-liver axis is mainly dependent on four transport proteins (Figure 2) (Tranah et al., 2021). The first transporter mediates BAs to be reabsorbed by the apical sodium-dependent bile acid transporter (ASBT) on the apical brush border membrane of ileocytes, the second is organic solute transporter-α and -β (OSTα/β) which export BAs to the portal vein circulation through the basolateral membrane, and the third, sodium/taurocholate cotransporting polypeptide (NTCP) takes up BAs from the portal vein circulation into the hepatocytes, while the bile salt export pump (BSEP) secretes BAs from the hepatocytes into the bile duct (Kunst et al., 2021).

#### 2.3.2 Enterohepatic circulation of BAs

The enterohepatic circulation is a very important BAs recycling system, most of the BAs molecules (about 95%) are reabsorbed daily into the circulating BAs pool, this reabsorption occurs mainly in the ileum and only a small fraction (about 5%) is excreted into the feces (Figure 2) (Chiang, 2013).

In the distal ileum, BAs are reabsorbed by ASBT into enterocytes, where BAs can activate FXR and induce the expression of fibroblast growth factor 15/19 (FGF15/19). Mouse FGF15 and human FGF19 have been considered as BAs induced intestinal factors, which are released to the portal vein and then circulated to hepatocytes to activate the FGF receptor 4 (FGFR4)/b-Klotho complex. By activating the activation of c-Jun N-terminal kinase dependent pathway of mitogen activated protein kinase (MAPK) pathway, they inhibit the expression of CYP7A1, reduce the synthesis of BAs in the liver and affect the size of BAs pool (Inagaki et al., 2005; Inagaki et al., 2006). The decrease of intraluminal BAs in patients with cholestatic liver disease can lead to the upregulation of ASBT; when negative feedback regulation occurs, it will cause the gradual increase of BAs in hepatocytes, leading to hepatocyte necrosis or apoptosis (Hofmann, 1999). Results of a study in chronic cholestasis (Mdr2 knockout) mice showed that inhibition of ASBT improved liver injury and reduced the expression of pro-inflammatory and pro-fibrotic genes associated with BAs toxicity (Baghdasaryan et al., 2016). Although BA inhibits ASBT, upregulation of ileal bile acid binding protein (IBABP) in the intestine more readily causes BAs to bind to this protein and protects hepatocytes from BAs toxicity, thereby enabling BA transcellular membrane transport to the basolateral membrane. FXR acting on the basement membrane can act as a bile acid sensor to induce OSTα/β secretion of BAs into the portal circulation and can regulate BAs reabsorption and secretion in portal blood (Chiang and Ferrell, 2020). Research shows that OST α/β not only plays an important role in maintaining bile acid homeostasis, but also prevents cholestasis in intestinal cells and protects the integrity of intestinal barrier function (Ferrebee et al., 2018). In addition, on the basement membrane of hepatocytes, NTCP is the main BAs transporter. BAs inhibit the expression of NTCP and reduces the reabsorption of BAs outside the base. This regulatory mechanism can protect hepatocytes from cholestasis (Kunst et al., 2021).

There are two main pathways of feedback inhibition of BAs synthesis by FXR, one is that BAs activate intestinal FXR-FGF15/19 signal and inhibits CYP7A1 in hepatocytes. Another way is that in hepatocytes, FXR activation induces the expression of a small heterodimer partner (SHP) and inhibits the transcription of CYP8B1 and CYP7A1 genes in hepatocytes. The intestinal FXR-FGF15/19 signal of SHP mainly inhibits CYP7A1, while the hepatic FXR-SHP signal mainly inhibits CYP8B1 (Kong et al., 2012). FXR activates and induces BSEP in hepatocytes, so that BAs flow from hepatocytes to the bile duct. If bile secretion is damaged, it will lead to cholestasis, bile salt and other toxic components accumulate in hepatocytes and blood, lead to hepatotoxicity, cause inflammation and oxidative stress in hepatocytes, and significantly affect biliary fibrosis and liver cirrhosis (Trauner and Boyer, 2003; Yang et al., 2019). For example, progressive familial intrahepatic cholestasis (PFIC) is an inherited liver disease from infancy. It is characterized by cholestasis and jaundice, and gradually develops into cirrhosis, liver failure, hepatocellular carcinoma, and death. In patients with progressive familial intrahepatic cholestasis type 2 (PFIC2), BSEP gene mutation impaired bile salt secretion in the bile duct, resulting in cholestasis (Jansen et al., 1999; Imagawa et al., 2017). Released from the intestine, BAs in the portal vein circulation are reabsorbed back to the liver, and BAs is recycled and secreted into bile, and then reaches the intestine again. Therefore, the whole process of BAs secretion from the liver to the intestine and recycling is called “enterohepatic circulation”.

## 3 Bidirectional regulation of bile acids and intestinal microbiota

The human gut acts like an anaerobic bioreactor and serves as a habitat for most of the microorganisms in our intestinal tract. These microorganisms include various types of bacteria, archaea, and eukaryotes (Bäckhed et al., 2005), they have symbiotic or antagonistic effect the body. Human intestinal flora is composed of trillions of microorganisms. They belong to four main phyla: Firmicutes (60%), *Bacteroides* (22%), Actinobacteria (17%) and Proteobacteria (1%) (Ikegami and Honda, 2018). Environmental factors such as diet, alcohol and will disturb the structure of intestinal microbiota. The homeostasis of intestinal microbiota is affected by BAs, diet, some drugs (such as probiotics and antibiotics) and diseases. The imbalance of Microecology will have a negative impact on host metabolism and lead to alcoholic or non-alcoholic fatty liver, primary biliary cirrhosis, hepatocellular carcinoma, inflammatory bowel disease, and other diseases. The degree of imbalance will also strengthen and continue with liver disease (Vassallo et al., 2015).

### 3.1 Regulation of gut microbiota on bile acids

Intestinal microbiota participates in the synthesis of BAs and the cycle of its biological signals. On the contrary, BAs also fundamentally shape the intestinal microbiota. Bacteria BSH and 7α-dehydroxylase is very important to regulate the metabolism and balance of BAs. Almost all primary BAs process 7α-dehydroxylation are converted to secondary BAs, BSH and bile acid 7α-dehydroxylation reactionappearsr to be restricted to a limited number of intestinal anaerobes, accounting for a small part of the total flora of the colon. BSH is produced in humans by many Gram-positive and Gram-negative symbiotic bacteria in the intestinal microflora, including *Bacteroides*, *Clostridium*, *Lactobacillus*, Bifidobacterium and *Listeria* (Ridlon et al., 2016; Staley et al., 2017). In addition, BSH is considered to protect symbiotic bacteria from bile salt toxicity, contribute to bacterial survival and intestinal colonization, protect the intestine, and provide nutritional sources of sulfur, nitrogen and carbon for bacteria (Van Eldere et al., 1996; De Boever et al., 2000).

### 3.2 Regulation of bile acids on gut microbiota

In addition to its digestive function and direct antibacterial effect, BAs can also affect the intestinal environment, with secondary BAs causing bacterial membrane instability and increased intestinal permeability (Islam et al., 2011; Lachar and Bajaj, 2016). When the composition of secondary BAs secreted into the intestinal cavity changes, it will affect the structure and metabolism of intestinal flora, which is conducive to the growth of pathogenic bacteria (Bajaj, 2014). On the contrary, intestinal microbiota can not only deconjugates and modify primary bile acids to secondary BAs, but also inhibit the synthesis of BAs in liver by regulating FXR-FGF19 signal in ileum (Sayin et al., 2013; Chiang and Ferrell, 2020). Therefore, in liver cirrhosis, CYP7A1 is inhibited, the synthesis of classic pathway of BAs is reduced, and alternative pathway has become the main pathway of BAs synthesis (Ridlon et al., 2013).

Through the comparative analysis between germ-free (GF) and conventionally fed FXR −/− mice, the results showed that the increase of MCA level in GF mice explained the decrease of FXR dependent gene signal in ileum and BAs excretion in feces of GF mice. In the presence of intestinal flora, the decrease of BAs pool in mice may also reflect the decrease of bile acid reabsorption in the distal ileum and the increase of BAs excretion in feces (Claus et al., 2008). In the analysis of probiotic LGG in the treatment of mice with hepatic fibrosis induced by bile duct ligation and multidrug resistance protein 2 gene knockout, it is concluded that the therapeutic effect of LGG is to suppression of BAs *de novo* synthesis by upregulating intestinal FXR-FGF19 signal transduction, enhance the levels of Firmicutes and Actinomycetes with BSH activity, increase the excretion of BAs from feces, to prevent excessive liver injury and fibrosis caused by excessive toxic hepatic bile acids (Liu et al., 2020). Intestinal microorganisms are affected by the size and composition of BAs pool, and BAs can be used as an indicator of the severity of liver disease (Shao et al., 2021). In conclusion, the homeostasis of bile acid-intestinal microecology axis plays an important role in liver inflammation, fibrosis and liver cirrhosis.

## 4 Triangular relationship among liver cirrhosis, bile acids and intestinal microorganisms

Liver cirrhosis is the end stage of chronic liver disease. “Decompensated cirrhosis” is the more advanced stage of chronic liver disease, which is characterized by variceal bleeding, ascites, HE spontaneous bacterial peritonitis (SBP) or jaundice. Patients without these complications are called “compensated” cirrhosis (D'Amico et al., 2006). There is a direct relationship between the frequency and severity of complications in decompensated cirrhosis, such as bacterial infections and encephalopathy, as well as ecological imbalances and intestinal barrier dysfunction.

### 4.1 Changes in bile acid levels and impaired intestinal barrier during liver cirrhosis

Studies have shown that with the progress of chronic liver disease or liver cirrhosis, the concentration of total bile acids and the proportion of secondary BAs/primary BAs decrease significantly, and the concentration of serum BAs is significantly lower than that of feces. The levels of conjugation BAs and deconjugation primary BAs in serum were higher. Alcohol consumption is one of the causes of cirrhosis. In severe alcoholic liver cirrhosis with alcoholic hepatitis, serum CDCA increases relatively and overflows into the systemic circulation, while DCA decreases relatively (Kakiyama et al., 2013; Ciocan et al., 2018). Genta Kakiyama et al. (Kakiyama et al., 2014) Proposed that the total fecal and secondary fecal BAs concentrations of patients with cirrhosis with long-term alcohol consumption were significantly increased compared with the healthy control group of abstinence cirrhosis and other chronic liver diseases and non alcohol consumption. An analysis of BAs in patients with alcoholic hepatitis found that serum total BAs, conjugation BAs and serum FGF19 levels increased, but *de novo* BAs synthesis was inhibited. Importantly, this was positively correlated with alcohol-related Model for End-stage Liver Disease (Brandl et al., 2018).

Intestinal disorders lay the foundation for the disruption of the intestinal barrier in liver cirrhosis. Changes in gut microbiota and bacterial overgrowth have been recognized in clinical and animal models of liver cirrhosis (Shah et al., 2017). The severity of intestinal barrier dysfunction and intestinal bacterial translocation is related to the severity of liver cirrhosis, and is more severe in patients with ascites. Portal hypertension, changes in gut microbiota, as well as inflammation and oxidative stress, can affect gut barrier function and may lead to the occurrence of complications in liver cirrhosis (Albillos et al., 2022). Due to changes in the function and anatomy of intestinal permeability in patients with cirrhosis, bacterial overgrowth and imbalance lead to more and more bacterial species and microbial derived PAMPs migrating from the intestinal cavity to the systemic circulation or liver along the lymphatic and portal vein pathways, and gradually develop into a systemic inflammatory state (Albillos et al., 2020). The destruction of the intestinal barrier in cirrhosis includes the reduction of the secretion of antibacterial peptides by intestinal Paneth cell, such as α- Defensin, especially α- Defensin 5 and 7, which have been proved in human small intestinal tissues and experimental models of decompensated cirrhosis (Kaliannan, 2018). Interestingly, under the action of signals derived from intestinal microbiota, such as Lipopolysaccharides (LPS) and mural dipeptide, Paneth cell also secrete angiogenic molecules, promote intestinal and mesenteric angiogenesis, and promote the development of portal hypertension (Hassan et al., 2020).

### 4.2 During liver cirrhosis, the microecology gradually tends to be unbalanced, the pathogenic bacteria are enriched, and the local flora is reduced

BAs, as an important factor regulating intestinal microecology, elevated intraluminal BAs concentrations favor the growth of bacteria undergoing 7α-dehydroxylation, preventing ecological dysregulation and inflammatory marker release. In contrast, lower intraluminal BAs levels favor the growth of Gram-negative bacteria, which can lead to overgrowth of pathogenic bacteria and induce the release of inflammatory markers as well as an increased inflammatory response in the liver, and in cirrhosis, ecological dysregulation is the result of lower concentrations of bile salts entering the intraluminal (Islam et al., 2011; Ridlon et al., 2013). Recently, the term “cirrhosis dysbiosis ratio” indicates that microecological imbalance is an indicator related to the progression of liver cirrhosis (Bajaj et al., 2014). Compared with the healthy control group, changes in the proportion of microbiota in liver cirrhosis were observed. Potentially pathogenic bacterias (*Enterobacteriaceae* and *Streptococcaceae*) were common in patients with liver cirrhosis at the family level. *Proteobacteria* and *Fusobacteria* were relatively overgrown and the proportion of beneficial *Bacteroides*, *Lactobacillus* and *Bifidobacterium* was significantly reduced. These changes become more serious with the progression of liver cirrhosis. When progressing to HE, the gut microbiota dysbiosis may occur (Chen et al., 2011).

In a study, 454 pyrophosphate sequencing of the 16S ribosomal RNA V3 region with real-time quantitative polymerase chain reaction (qPCR) was performed to observe changes in the characteristics of the fecal microbial community in patients with liver cirrhosis. *Bacteroides* plays a dominant role in human intestinal microecology. The reduction of the microbiome of liver cirrhosis and *Lactobacillaceae* and *Bacteroidetes* may be related to the increase of Proteobacteria and Clostridia (most Proteobacterial sequences belong to the class of Aspergillus). It was proved that the child Turcotte Pugh (CTP) score was positively correlated with *Streptococcaceae*, and the significant reduction of *Clostridium* resulted in pro-inflammatory response, which was negatively correlated with CTP score (Chen et al., 2011; Gómez-Hurtado et al., 2011). In addition, the dramatic shift of the proportion of *Firmicutes*, particularly the *Clostridium* cluster XIVa, which stemmed from elevated CA levels and led to elevated DCA levels (Ridlon et al., 2013). In a study of a rat model of carbon tetrachloride-induced cirrhosis, fecal intestinal aerobic bacterial flora (e.g., E*scherichia coli*, *Proteobacteria*) were found to be enriched in the cecum (Guarner et al., 1997). The prevalence of *Streptococcus* and *Enterobacter* among the microorganisms in the stool of cirrhotic patients explains exactly why *Escherichia coli* and *Streptococcus* are the main causes of bacterial infections in cirrhotic patients (Riordan and Williams, 2006).

### 4.3 Hepatic encephalopathy under the influence of BAs and gut microbiota

HE is a common neuropsychiatric complication characterized by hyperammonemia in the course of cirrhosis. It has previously been proved to be related to the increase of ammonia and BAs levels in the blood (Riordan and Williams, 1997; Munoz, 2008). Blood ammonia mainly comes from the intestine. Excessive ammonia produced by intestinal bacteria may lead to the increase of ammonia content in the circulation. At the same time, liver damage leads to the disorder of urea circulation. Insufficient ammonia clearance can also lead to the abnormal increase of ammonia concentration in the circulation. Ammonia is easy to enter the brain through the blood-brain barrier (BBB) (Adlimoghaddam et al., 2016). The serum conjugated BAs of patients with cirrhosis increases. The further increase of BAs in the blood during HE will lead to the increase of brain bile acid. Because BAs can be used as a detergent, it can induce the permeability of blood-brain barrier and lead to brain injury (Figure 2) (Li and Chiang, 2015). In a mouse model of chronic liver disease and acute liver failure (ALF) induced by azomethane (AMO), ASBT mediated BAs reabsorption, increased the pH value of intestinal cavity, increased the amount of ammonia, promoted the conversion of intestinal ammonia into blood ammonia, resulting in abnormally high levels of neurotoxic ammonia and cytotoxic BAs in blood and brain, and finally damaged nerves. In contrast, SC-435 (ASBT inhibitor) blocks intestinal bile acid reabsorption and reduces circulating BAs and ammonia concentrations, thereby reducing liver and brain damage (Xie et al., 2018). Accordingly, ASBT inhibitors may become a new treatment method, predominantly for chronic neurological diseases dominated by BAs and ammonia regulation disorders, such as HE.

Altered intestinal microbiome and intestinal permeability may occur with associated dysfunctional bidirectional brain and intestinal actions (Vilstrup et al., 2014). The clinical classification of HE ranges from mild or recessive HE (MHE) to overt HE (OHE) with progressive cognitive decline in patients, the pathophysiological mechanisms of which are unclear and are currently proposed to be possibly related to ammonia accumulation and gut microbial activity. Cirrhosis in GF mice exhibits hyperammonemia, but this is not associated with systemic or neuroinflammation, and these inflammatory responses are only seen in conventionally housed cirrhotic mice (Kang et al., 2016). In patients with cirrhosis, urease containing microorganisms are an important source of ammonia to the body, usually Gram-negative bacteria such as *Streptococcus salivarius* and *Proteobacteria*. They catalyze the conversion of urea to ammonia. However, impaired liver clearance leads to ammonia accumulation and in synergy with inflammation exacerbates nerve damage triggering HE (Yukawa-Muto et al., 2022). This illustrates the central role of the gut microbiota in the pathogenesis of human HE and shows evidence of neuroinflammation, systemic inflammation, hyperammonemia and cerebral edema.

Altered microbiota function is likely to be mechanistically related to cognitive impairment as confirmed by rifaximin, an effective antibiotic for HE with broad-spectrum antimicrobial activity, and reduced endotoxemia and improved cognitive function in its treatment of patients with cirrhosis and MHE, accompanied by reduced harmful metabolites, altered BAs composition and intestinal microecology (Bajaj et al., 2013; Dalal et al., 2017). *Enterobacteriaceae* positively correlated with fecal CDCA in humans and were strongly associated with the development of HE, while *Ruminococcaceae* positively correlated with DCA (Kakiyama et al., 2013). DCA is the most effective bactericide for controlling intestinal bacterial growth and intestinal microbes, and the improvement in cognitive function and endotoxemia in HE patients is also the result of a lower DCA/CA ratio (Ridlon et al., 2013). Several studies below describe more specifically the link between microecological dysregulation and HE. Significant disruption of the intestinal microecological flora in patients with MHE, with overgrowth of potentially pathogenic *Escherichia coli* and *Staphylococcus spp* (Liu et al., 2004). Other studies have shown that specific bacterial groups in patients with cirrhotic HE, including the alkali-producing *Bacillariophyceae*, *Porphyromonas* and *Enterobacteriaceae* are directly related to poorer cognitive performance and increased inflammation in OHE. In addition, *Alcaligenaceae* and *Escherichia coli* were significantly more frequent in cirrhotic patients with HE compared to the group without HE, and *Alcaligenaceae* and *Porphyromonadaceae* were positively associated with cognitive impairment, whereas *Fusobacteriaceae*, *Enterobacteriaceae* and *Veillonellaceae* were positively associated with inflammation (Bajaj et al., 2012a; Bajaj et al., 2012b). These suggest that interactions between inflammation, cognitive function, and microbiota changes play a role in HE and may predict the development of HE (Bajaj et al., 2019).

## 5 Conclusion

BAs, a center factor in the regulation of the intestine-liver-brain axis, has a beneficial effect on BAs homeostasis and microbiota to some extent, but it also affects the permeability of the intestinal barrier and the blood-brain barrier, which can cause complications such as HE. Altered gut microbiota diversity and disturbed BAs metabolism in cirrhosis have led to a focus on key pathological mechanisms of dysbiosis and altered barrier permeability as potential targets and approaches for the treatment of cirrhosis. More personalized treatment strategies, such as the use of targeted probiotics, antibiotics, and fecal transplants, can improve cirrhosis, hepatic cholestasis status, and neurological function of the brain by modulating specific flora to affect bile acid composition. In conclusion, a deeper understanding of the complex relationship between BAs signaling and microecology is expected to help us use this knowledge in the clinical setting to improve future strategies for the treatment of cirrhosis.

## References

[B1] Adlimoghaddam A. Sabbir M. G. Albensi B. C. (2016). Ammonia as a potential neurotoxic factor in alzheimer's disease. Front. Mol. Neurosci. 9, 57. 10.3389/fnmol.2016.00057 27551259 PMC4976099

[B2] Albillos A. De Gottardi A. Rescigno M. (2020). The gut-liver axis in liver disease: Pathophysiological basis for therapy. J. Hepatology 72, 558–577. 10.1016/j.jhep.2019.10.003 31622696

[B3] Albillos A. Martin-Mateos R. Van Der Merwe S. Wiest R. Jalan R. Álvarez-Mon M. (2022). Cirrhosis-associated immune dysfunction. Nat. Rev. Gastroenterology Hepatology 19, 112–134. 10.1038/s41575-021-00520-7 34703031

[B4] Axelson M. Aly A. Sjövall J. (1988). Levels of 7 alpha-hydroxy-4-cholesten-3-one in plasma reflect rates of bile acid synthesis in man. FEBS Lett. 239, 324–328. 10.1016/0014-5793(88)80944-x 3181435

[B5] Bäckhed F. Ley R. E. Sonnenburg J. L. Peterson D. A. Gordon J. I. (2005). Host-bacterial mutualism in the human intestine. Sci. (New York, N.Y.) 307, 1915–1920. 10.1126/science.1104816 15790844

[B6] Baghdasaryan A. Fuchs C. D. Österreicher C. H. Lemberger U. J. Halilbasic E. Påhlman I. (2016). Inhibition of intestinal bile acid absorption improves cholestatic liver and bile duct injury in a mouse model of sclerosing cholangitis. J. hepatology 64, 674–681. 10.1016/j.jhep.2015.10.024 26529078

[B7] Bajaj J. S. Gillevet P. M. Patel N. R. Ahluwalia V. Ridlon J. M. Kettenmann B. (2012a). A longitudinal systems biology analysis of lactulose withdrawal in hepatic encephalopathy. Metab. Brain Dis. 27, 205–215. 10.1007/s11011-012-9303-0 22527995

[B8] Bajaj J. S. Heuman D. M. Hylemon P. B. Sanyal A. J. White M. B. Monteith P. (2014). Altered profile of human gut microbiome is associated with cirrhosis and its complications. J. hepatology 60, 940–947. 10.1016/j.jhep.2013.12.019 PMC3995845 24374295

[B9] Bajaj J. S. Heuman D. M. Sanyal A. J. Hylemon P. B. Sterling R. K. Stravitz R. T. (2013). Modulation of the metabiome by rifaximin in patients with cirrhosis and minimal hepatic encephalopathy. PloS One 8, e60042. 10.1371/journal.pone.0060042 23565181 PMC3615021

[B10] Bajaj J. S. Ridlon J. M. Hylemon P. B. Thacker L. R. Heuman D. M. Smith S. (2012b). Linkage of gut microbiome with cognition in hepatic encephalopathy. Am. J. Physiol. Gastrointest. Liver Physiol. 302, G168–G175. 10.1152/ajpgi.00190.2011 21940902 PMC3345956

[B11] Bajaj J. S. (2014). The role of microbiota in hepatic encephalopathy. Gut microbes 5, 397–403. 10.4161/gmic.28684 24690956 PMC4153779

[B12] Bajaj J. S. Vargas H. E. Reddy K. R. Lai J. C. O'leary J. G. Tandon P. (2019). Association between intestinal microbiota collected at hospital admission and outcomes of patients with cirrhosis. Clin. Gastroenterol. Hepatol. 17, 756–765. 10.1016/j.cgh.2018.07.022 30036646

[B13] Begley M. Gahan C. G. Hill C. (2005). The interaction between bacteria and bile. FEMS Microbiol. Rev. 29, 625–651. 10.1016/j.femsre.2004.09.003 16102595

[B14] Brandl K. Hartmann P. Jih L. J. Pizzo D. P. Argemi J. Ventura-Cots M. (2018). Dysregulation of serum bile acids and FGF19 in alcoholic hepatitis. J. hepatology 69, 396–405. 10.1016/j.jhep.2018.03.031 PMC6054564 29654817

[B15] Chen Y. Yang F. Lu H. Wang B. Chen Y. Lei D. (2011). Characterization of fecal microbial communities in patients with liver cirrhosis. Hepatology 54, 562–572. 10.1002/hep.24423 21574172

[B16] Chiang J. Y. L. (2013). Bile acid metabolism and signaling. Compr. Physiol. 3, 1191–1212. 10.1002/cphy.c120023 23897684 PMC4422175

[B17] Chiang J. Y. L. (2009). Bile acids: Regulation of synthesis. J. lipid Res. 50, 1955–1966. 10.1194/jlr.R900010-JLR200 19346330 PMC2739756

[B18] Chiang J. Y. L. Ferrell J. M. (2020). Bile acid receptors FXR and TGR5 signaling in fatty liver diseases and therapy. Am. J. physiology. Gastrointest. liver physiology 318, G554–G573. 10.1152/ajpgi.00223.2019 PMC7099488 31984784

[B19] Chiang J. Y. L. (2004). Regulation of bile acid synthesis: Pathways, nuclear receptors, and mechanisms. J. hepatology 40, 539–551. 10.1016/j.jhep.2003.11.006 15123373

[B20] Ciocan D. Voican C. S. Wrzosek L. Hugot C. Rainteau D. Humbert L. (2018). Bile acid homeostasis and intestinal dysbiosis in alcoholic hepatitis. Aliment. Pharmacol. Ther. 48, 961–974. 10.1111/apt.14949 30144108

[B21] Claus S. P. Tsang T. M. Wang Y. Cloarec O. Skordi E. Martin F.-P. (2008). Systemic multicompartmental effects of the gut microbiome on mouse metabolic phenotypes. Mol. Syst. Biol. 4, 219. 10.1038/msb.2008.56 18854818 PMC2583082

[B22] D'amico G. Garcia-Tsao G. Pagliaro L. (2006). Natural history and prognostic indicators of survival in cirrhosis: A systematic review of 118 studies. J. Hepatol. 44, 217–231. 10.1016/j.jhep.2005.10.013 16298014

[B23] Dalal R. Mcgee R. G. Riordan S. M. Webster A. C. (2017). Probiotics for people with hepatic encephalopathy. Cochrane Database Syst. Rev. 2, CD008716. 10.1002/14651858.CD008716.pub3 28230908 PMC6464663

[B24] De Boever P. Wouters R. Verschaeve L. Berckmans P. Schoeters G. Verstraete W. (2000). Protective effect of the bile salt hydrolase-active Lactobacillus reuteri against bile salt cytotoxicity. Appl. Microbiol. Biotechnol. 53, 709–714. 10.1007/s002530000330 10919331

[B25] Eyssen H. De Pauw G. Stragier J. Verhulst A. (1983). Cooperative formation of omega-muricholic acid by intestinal microorganisms. Appl. Environ. Microbiol. 45, 141–147. 10.1128/AEM.45.1.141-147.1983 6824314 PMC242244

[B26] Fakheri R. J. Javitt N. B. (2012). 27-Hydroxycholesterol, does it exist? On the nomenclature and stereochemistry of 26-hydroxylated sterols. Steroids 77, 575–577. 10.1016/j.steroids.2012.02.006 22366074

[B27] Ferrebee C. B. Li J. Haywood J. Pachura K. Robinson B. S. Hinrichs B. H. (2018). Organic solute transporter α-β protects ileal enterocytes from bile acid-induced injury. Cell. Mol. gastroenterology hepatology 5, 499–522. 10.1016/j.jcmgh.2018.01.006 PMC6009794 29930976

[B28] Fiorucci S. Rizzo G. Antonelli E. Renga B. Mencarelli A. Riccardi L. (2005). Cross-talk between farnesoid-X-receptor (FXR) and peroxisome proliferator-activated receptor gamma contributes to the antifibrotic activity of FXR ligands in rodent models of liver cirrhosis. J. Pharmacol. Exp. Ther. 315, 58–68. 10.1124/jpet.105.085597 15980055

[B29] Fu Z. D. Csanaky I. L. Klaassen C. D. (2012). Gender-divergent profile of bile acid homeostasis during aging of mice. PloS one 7, e32551. 10.1371/journal.pone.0032551 22403674 PMC3293819

[B30] Gómez-Hurtado I. Santacruz A. Peiró G. Zapater P. Gutiérrez A. Pérez-Mateo M. (2011). Gut microbiota dysbiosis is associated with inflammation and bacterial translocation in mice with CCl4-induced fibrosis. PloS one 6, e23037. 10.1371/journal.pone.0023037 21829583 PMC3146520

[B31] Gruner N. Mattner J. (2021). Bile acids and microbiota: Multifaceted and versatile regulators of the liver-gut Axis. Int. J. Mol. Sci. 22, 1397. 10.3390/ijms22031397 33573273 PMC7866539

[B32] Guarner C. Runyon B. A. Young S. Heck M. Sheikh M. Y. (1997). Intestinal bacterial overgrowth and bacterial translocation in cirrhotic rats with ascites. J. hepatology 26, 1372–1378. 10.1016/s0168-8278(97)80474-6 9210626

[B33] Hassan M. Moghadamrad S. Sorribas M. Muntet S. G. Kellmann P. Trentesaux C. (2020). Paneth cells promote angiogenesis and regulate portal hypertension in response to microbial signals. J. Hepatology 73, 628–639. 10.1016/j.jhep.2020.03.019 32205193

[B34] Heverin M. Meaney S. Lütjohann D. Diczfalusy U. Wahren J. Björkhem I. (2005). Crossing the barrier: Net flux of 27-hydroxycholesterol into the human brain. J. lipid Res. 46, 1047–1052. 10.1194/jlr.M500024-JLR200 15741649

[B35] Hofmann A. F. (1984). Chemistry and enterohepatic circulation of bile acids. Hepatology 4, 4S–14S. 10.1002/hep.1840040803 6384004

[B36] Hofmann A. F. (1999). Regulation of ileal bile acid transport: Desirability of measuring transport function as well as transporter activity. Hepatology 29, 1335–1337. 10.1002/hep.510290430 10336336

[B37] Ikegami T. Honda A. (2018). Reciprocal interactions between bile acids and gut microbiota in human liver diseases. Hepatology Res. official J. Jpn. Soc. Hepatology 48, 15–27. 10.1111/hepr.13001 29150974

[B38] Imagawa K. Takayama K. Isoyama S. Tanikawa K. Shinkai M. Harada K. (2017). Generation of a bile salt export pump deficiency model using patient-specific induced pluripotent stem cell-derived hepatocyte-like cells. Sci. Rep. 7, 41806. 10.1038/srep41806 28150711 PMC5288783

[B39] Inagaki T. Choi M. Moschetta A. Peng L. Cummins C. L. Mcdonald J. G. (2005). Fibroblast growth factor 15 functions as an enterohepatic signal to regulate bile acid homeostasis. Cell Metab. 2, 217–225. 10.1016/j.cmet.2005.09.001 16213224

[B40] Inagaki T. Moschetta A. Lee Y.-K. Peng L. Zhao G. Downes M. (2006). Regulation of antibacterial defense in the small intestine by the nuclear bile acid receptor. Proc. Natl. Acad. Sci. U. S. A. 103, 3920–3925. 10.1073/pnas.0509592103 16473946 PMC1450165

[B41] Islam K. B. Fukiya S. Hagio M. Fujii N. Ishizuka S. Ooka T. (2011). Bile acid is a host factor that regulates the composition of the cecal microbiota in rats. Gastroenterology 141, 1773–1781. 10.1053/j.gastro.2011.07.046 21839040

[B42] Jansen P. L. Strautnieks S. S. Jacquemin E. Hadchouel M. Sokal E. M. Hooiveld G. J. (1999). Hepatocanalicular bile salt export pump deficiency in patients with progressive familial intrahepatic cholestasis. Gastroenterology 117, 1370–1379. 10.1016/s0016-5085(99)70287-8 10579978

[B43] Jelinek D. F. Andersson S. Slaughter C. A. Russell D. W. (1990). Cloning and regulation of cholesterol 7 alpha-hydroxylase, the rate-limiting enzyme in bile acid biosynthesis. J. Biol. Chem. 265, 8190–8197. 10.1016/s0021-9258(19)39056-8 2335522 PMC4451855

[B44] Kakiyama G. Hylemon P. B. Zhou H. Pandak W. M. Heuman D. M. Kang D. J. (2014). Colonic inflammation and secondary bile acids in alcoholic cirrhosis. Am. J. physiology. Gastrointest. liver physiology 306, G929–G937. 10.1152/ajpgi.00315.2013 PMC4152166 24699327

[B45] Kakiyama G. Pandak W. M. Gillevet P. M. Hylemon P. B. Heuman D. M. Daita K. (2013). Modulation of the fecal bile acid profile by gut microbiota in cirrhosis. J. Hepatol. 58, 949–955. 10.1016/j.jhep.2013.01.003 23333527 PMC3936319

[B46] Kaliannan K. (2018). Compromise of α-defensin function in liver cirrhosis facilitates the toxic relationship between gut permeability and endotoxemia. Dig. Dis. Sci. 63, 2492–2494. 10.1007/s10620-018-5197-y 30008088

[B47] Kang D. J. Betrapally N. S. Ghosh S. A. Sartor R. B. Hylemon P. B. Gillevet P. M. (2016). Gut microbiota drive the development of neuroinflammatory response in cirrhosis in mice. Hepatology 64, 1232–1248. 10.1002/hep.28696 27339732 PMC5033692

[B48] Kiriyama Y. Nochi H. (2019). The biosynthesis, signaling, and neurological functions of bile acids. Biomolecules 9, 232. 10.3390/biom9060232 31208099 PMC6628048

[B49] Kok T. Hulzebos C. V. Wolters H. Havinga R. Agellon L. B. Stellaard F. (2003). Enterohepatic circulation of bile salts in farnesoid X receptor-deficient mice: Efficient intestinal bile salt absorption in the absence of ileal bile acid-binding protein. J. Biol. Chem. 278, 41930–41937. 10.1074/jbc.M306309200 12917447

[B50] Kong B. Wang L. Chiang J. Y. L. Zhang Y. Klaassen C. D. Guo G. L. (2012). Mechanism of tissue-specific farnesoid X receptor in suppressing the expression of genes in bile-acid synthesis in mice. Hepatology 56, 1034–1043. 10.1002/hep.25740 22467244 PMC3390456

[B51] Kunst R. F. Verkade H. J. Oude Elferink R. P. J. Van De Graaf S. F. J. (2021). Targeting the four pillars of enterohepatic bile salt cycling; lessons from genetics and Pharmacology. Hepatology 73, 2577–2585. 10.1002/hep.31651 33222321 PMC8252069

[B52] Lachar J. Bajaj J. S. (2016). Changes in the microbiome in cirrhosis and relationship to complications: Hepatic encephalopathy, spontaneous bacterial peritonitis, and sepsis. Semin. Liver Dis. 36, 327–330. 10.1055/s-0036-1593881 27997972

[B53] Li T. Apte U. (2015). Bile acid metabolism and signaling in cholestasis, inflammation, and cancer. Adv. Pharmacol. 74, 263–302. 10.1016/bs.apha.2015.04.003 26233910 PMC4615692

[B54] Li T. Chiang J. Y. (2014). Bile acid signaling in metabolic disease and drug therapy. Pharmacol. Rev. 66, 948–983. 10.1124/pr.113.008201 25073467 PMC4180336

[B55] Li T. Chiang J. Y. L. (2015). Bile acids as metabolic regulators. Curr. Opin. gastroenterology 31, 159–165. 10.1097/MOG.0000000000000156 PMC4332523 25584736

[B56] Liu Q. Duan Z. P. Ha D. K. Bengmark S. Kurtovic J. Riordan S. M. (2004). Synbiotic modulation of gut flora: Effect on minimal hepatic encephalopathy in patients with cirrhosis. Hepatology 39, 1441–1449. 10.1002/hep.20194 15122774

[B57] Liu Y. Chen K. Li F. Gu Z. Liu Q. He L. (2020). Probiotic Lactobacillus rhamnosus GG prevents liver fibrosis through inhibiting hepatic bile acid synthesis and enhancing bile acid excretion in mice. Hepatology 71, 2050–2066. 10.1002/hep.30975 31571251 PMC7317518

[B58] Lund E. G. Xie C. Kotti T. Turley S. D. Dietschy J. M. Russell D. W. (2003). Knockout of the cholesterol 24-hydroxylase gene in mice reveals a brain-specific mechanism of cholesterol turnover. J. Biol. Chem. 278, 22980–22988. 10.1074/jbc.M303415200 12686551

[B59] Mcmillin M. Frampton G. Quinn M. Ashfaq S. De Los Santos M. Grant S. (2016). Bile acid signaling is involved in the neurological decline in a murine model of acute liver failure. Am. J. Pathology 186, 312–323. 10.1016/j.ajpath.2015.10.005 PMC4729266 26683664

[B60] Munoz S. J. (2008). Hepatic encephalopathy. Med Clin North Am 92, 795–812. 10.1016/j.mcna.2008.03.009 18570943

[B61] Pineda Torra I. Claudel T. Duval C. Kosykh V. Fruchart J.-C. Staels B. (2003). Bile acids induce the expression of the human peroxisome proliferator-activated receptor alpha gene via activation of the farnesoid X receptor. Mol. Endocrinol. 17, 259–272. 10.1210/me.2002-0120 12554753

[B62] Ridlon J. M. Alves J. M. Hylemon P. B. Bajaj J. S. (2013). Cirrhosis, bile acids and gut microbiota: Unraveling a complex relationship. Gut Microbes 4, 382–387. 10.4161/gmic.25723 23851335 PMC3839982

[B63] Ridlon J. M. Harris S. C. Bhowmik S. Kang D.-J. Hylemon P. B. (2016). Consequences of bile salt biotransformations by intestinal bacteria. Gut microbes 7, 22–39. 10.1080/19490976.2015.1127483 26939849 PMC4856454

[B64] Riordan S. M. Williams R. (2006). The intestinal flora and bacterial infection in cirrhosis. J. hepatology 45, 744–757. 10.1016/j.jhep.2006.08.001 16979776

[B65] Riordan S. M. Williams R. (1997). Treatment of hepatic encephalopathy. N. Engl. J. Med. 337, 473–479. 10.1056/NEJM199708143370707 9250851

[B66] Russell D. W. Setchell K. D. (1992). Bile acid biosynthesis. Biochemistry 31, 4737–4749. 10.1021/bi00135a001 1591235

[B67] Sauerbruch T. Hennenberg M. Trebicka J. Beuers U. (2021). Bile acids, liver cirrhosis, and extrahepatic vascular dysfunction. Front. Physiol. 12, 718783. 10.3389/fphys.2021.718783 34393832 PMC8358446

[B68] Sayin S. I. Wahlstrom A. Felin J. Jantti S. Marschall H. U. Bamberg K. (2013). Gut microbiota regulates bile acid metabolism by reducing the levels of tauro-beta-muricholic acid, a naturally occurring FXR antagonist. Cell Metab. 17, 225–235. 10.1016/j.cmet.2013.01.003 23395169

[B69] Shah A. Shanahan E. Macdonald G. A. Fletcher L. Ghasemi P. Morrison M. (2017). Systematic review and meta-analysis: Prevalence of small intestinal bacterial overgrowth in chronic liver disease. Seminars Liver Dis. 37, 388–400. 10.1055/s-0037-1608832 29272899

[B70] Shao J. W. Ge T. T. Chen S. Z. Wang G. Yang Q. Huang C. H. (2021). Role of bile acids in liver diseases mediated by the gut microbiome. World J. Gastroenterol. 27, 3010–3021. 10.3748/wjg.v27.i22.3010 34168404 PMC8192287

[B71] Simbrunner B. Trauner M. Reiberger T. (2021). Review article: Therapeutic aspects of bile acid signalling in the gut-liver axis. Alimentary Pharmacol. Ther. 54, 1243–1262. 10.1111/apt.16602 PMC9290708 34555862

[B72] Sorribas M. Jakob M. O. Yilmaz B. Li H. Stutz D. Noser Y. (2019). FXR modulates the gut-vascular barrier by regulating the entry sites for bacterial translocation in experimental cirrhosis. J. Hepatology 71, 1126–1140. 10.1016/j.jhep.2019.06.017 31295531

[B73] Staley C. Weingarden A. R. Khoruts A. Sadowsky M. J. (2017). Interaction of gut microbiota with bile acid metabolism and its influence on disease states. Appl. Microbiol. Biotechnol. 101, 47–64. 10.1007/s00253-016-8006-6 27888332 PMC5203956

[B74] Stofan M. Guo G. L. (2020). Bile acids and FXR: Novel targets for liver diseases. Front. Med. 7, 544. 10.3389/fmed.2020.00544 PMC7516013 33015098

[B75] Tranah T. H. Edwards L. A. Schnabl B. Shawcross D. L. (2021). Targeting the gut-liver-immune axis to treat cirrhosis. Gut 70, 982–994. 10.1136/gutjnl-2020-320786 33060124

[B76] Trauner M. Boyer J. L. (2003). Bile salt transporters: Molecular characterization, function, and regulation. Physiol. Rev. 83, 633–671. 10.1152/physrev.00027.2002 12663868

[B77] Van Eldere J. Celis P. De Pauw G. Lesaffre E. Eyssen H. (1996). Tauroconjugation of cholic acid stimulates 7 alpha-dehydroxylation by fecal bacteria. Appl. Environ. Microbiol. 62, 656–661. 10.1128/AEM.62.2.656-661.1996 8593067 PMC167832

[B78] Vassallo G. Mirijello A. Ferrulli A. Antonelli M. Landolfi R. Gasbarrini A. (2015). Review article: Alcohol and gut microbiota - the possible role of gut microbiota modulation in the treatment of alcoholic liver disease. Alimentary Pharmacol. Ther. 41, 917–927. 10.1111/apt.13164 25809237

[B79] Vilstrup H. Amodio P. Bajaj J. Cordoba J. Ferenci P. Mullen K. D. (2014). Hepatic encephalopathy in chronic liver disease: 2014 practice guideline by the American association for the study of liver diseases and the European association for the study of the liver. Hepatol. Baltim. Md 60, 715–735. 10.1002/hep.27210 25042402

[B80] Xie G. Wang X. Jiang R. Zhao A. Yan J. Zheng X. (2018). Dysregulated bile acid signaling contributes to the neurological impairment in murine models of acute and chronic liver failure. EBioMedicine 37, 294–306. 10.1016/j.ebiom.2018.10.030 30344125 PMC6284422

[B81] Yang T. Khan G. J. Wu Z. Wang X. Zhang L. Jiang Z. (2019). Bile acid homeostasis paradigm and its connotation with cholestatic liver diseases. Drug Discov. Today 24, 112–128. 10.1016/j.drudis.2018.09.007 30244079

[B82] Yukawa-Muto Y. Kamiya T. Fujii H. Mori H. Toyoda A. Sato I. (2022). Distinct responsiveness to rifaximin in patients with hepatic encephalopathy depends on functional gut microbial species. Hepatol. Commun. 6, 2090–2104. 10.1002/hep4.1954 35429147 PMC9315133

